# The pitfalls of scaling up evidence-based interventions in health

**DOI:** 10.1080/16549716.2019.1670449

**Published:** 2019-10-02

**Authors:** Hervé Tchala Vignon Zomahoun, Ali Ben Charif, Adriana Freitas, Mirjam Marjolein Garvelink, Matthew Menear, Michèle Dugas, Rhéda Adekpedjou, France Légaré

**Affiliations:** a Health and Social Services Systems, Knowledge Translation and Implementation component of the Quebec SPOR-SUPPORT Unit, Université Laval, Quebec, QC, Canada; b Centre de recherche sur les soins et les services de première ligne – Université Laval (CERSSPL-UL), Université Laval, Quebec, QC, Canada; c Tier 1 Canada Research Chair in Shared Decision Making and Knowledge Translation, Université Laval, Quebec, QC, Canada; d Department of Family Medicine and Emergency Medicine, Université Laval, Quebec, QC, Canada; e Population Health and Practice-Changing Research Group, CHU de Québec Research Centre, Quebec, QC, Canada

**Keywords:** Scaling-up, evidence-based intervention, harms, equity, cost-effectiveness, ethics, health

## Abstract

Policy-makers worldwide are increasingly interested in scaling up evidence-based interventions (EBIs) to larger populations, and implementation scientists are developing frameworks and methodologies for achieving this. But scaling-up does not always produce the desired results. Why not? We aimed to enhance awareness of the various pitfalls to be anticipated when planning scale-up. In lower- and middle-income countries (LMICs), the scale-up of health programs to prevent or respond to outbreaks of communicable diseases has been occurring for many decades. In high-income countries, there is new interest in the scaling up of interventions that address communicable and non-communicable diseases alike. We scanned the literature worldwide on problems encountered when implementing scale-up plans revealed a number of potential pitfalls that we discuss in this paper. We identified and discussed the following six major pitfalls of scaling-up EBIs: 1) the cost-effectiveness estimation pitfall, i.e. accurate cost-effectiveness estimates about real-world implementation are almost impossible, making predictions of economies of scale unreliable; 2) the health inequities pitfall, i.e. some people will necessarily be left out and therefore not benefit from the scaled-up EBIs; 3) the scaled-up harm pitfall, i.e. the harms as well as the benefits may be amplified by the scaling-up; 4) the ethical pitfall, i.e. informed consent may be a challenge on a grander scale; 5) the top-down pitfall, i.e. the needs, preferences and culture of end-users may be forgotten when scale-up is directed from above; and 6) the contextual pitfall, i.e. it may not be possible to adapt the EBIs to every context. If its pitfalls are addressed head on, scaling-up may be a powerful process for translating research data into practical improvements in healthcare in both LMICs and high-income countries, ensuring that more people benefit from EBIs.

## Background

In lower- and middle-income countries (LMICs), the scale-up of health interventions is not a new concept, although it has been often called by other names. For many decades, through international and national agencies, small-scale interventions have been scaled up to larger populations in LMICs to prevent or respond to outbreaks of communicable diseases [1]. Preventable noncommunicable diseases are now becoming as much of a burden in LMICs as in high-income countries [2]. But not all scaling-up efforts produce the desired results. There is thus a shared interest in knowing the pitfalls of scaling-up, and addressing them on a global scale.

Evidence-based interventions (EBIs) are interventions that have been proven effective, efficacious and ready for dissemination [3]. For example, motivational interviewing is a reproducible intervention that has been shown to be effective for the primary and secondary prevention of disease by improving health behaviors such as medication adherence [4], smoking cessation [5], and physical activity [6]. However, this and other EBIs often stay at the research level and fail to reach the people who should benefit from them. To get better value for their investments in research, policy-makers are taking a new interest in scaling-up, defined by the World Health Organization (WHO) as ‘deliberate efforts to increase the impact of successfully tested health innovations so as to benefit more people and to foster policy and program development on a lasting basis’ [7,8].

Two examples of scaling up demonstrate very different processes. In Ghana, a successful community-based health services experiment was scaled up to 104 out of 110 districts [9]. The process included piloting, field demonstrations, resource assessment, leadership development, building and equipping facilities and assigning health staff, training counterparts and deploying volunteers. Authors credit its success to consensus building, a sense of ownership among the targeted communities, the presence of change agents, credibility of the change and demonstration of feasibility in the communities. They also credit the involvement of all levels of bureaucracy in the change.

In another example in British Columbia, Canada, a program to integrate physical education and healthy eating in schools was scaled up to reach 500,000 students and 81,000 teachers [10]. Scale-up involved establishing action zones, a central support team, school facilitators and stakeholder teams, a planning guide and resource directory, and bins filled with exercise equipment. This program credits its success at the micro-level to training and resourcing teachers, supportive school policies and sustained implementation. At the macro-level, they credit their success to multisectorial partnerships and embedded knowledge exchange mechanisms.

In high-income countries, the science of scaling-up is still in its infancy. Various frameworks have been proposed for guiding researchers and policy-makers in designing the scale-up of an EBI [7,11–19]. Researchers are also proposing methods for evaluating the scalability of interventions, using indicators such as coverage, impact and setting, to prepare researchers to include them in the earliest stages of their study designs [20]. While these frameworks appear convincing and the evolving discourse on scaling-up is enthusiastic, a brief scan of the literature for problems encountered during scale-up revealed a number of potential pitfalls.

**Figure 1.**
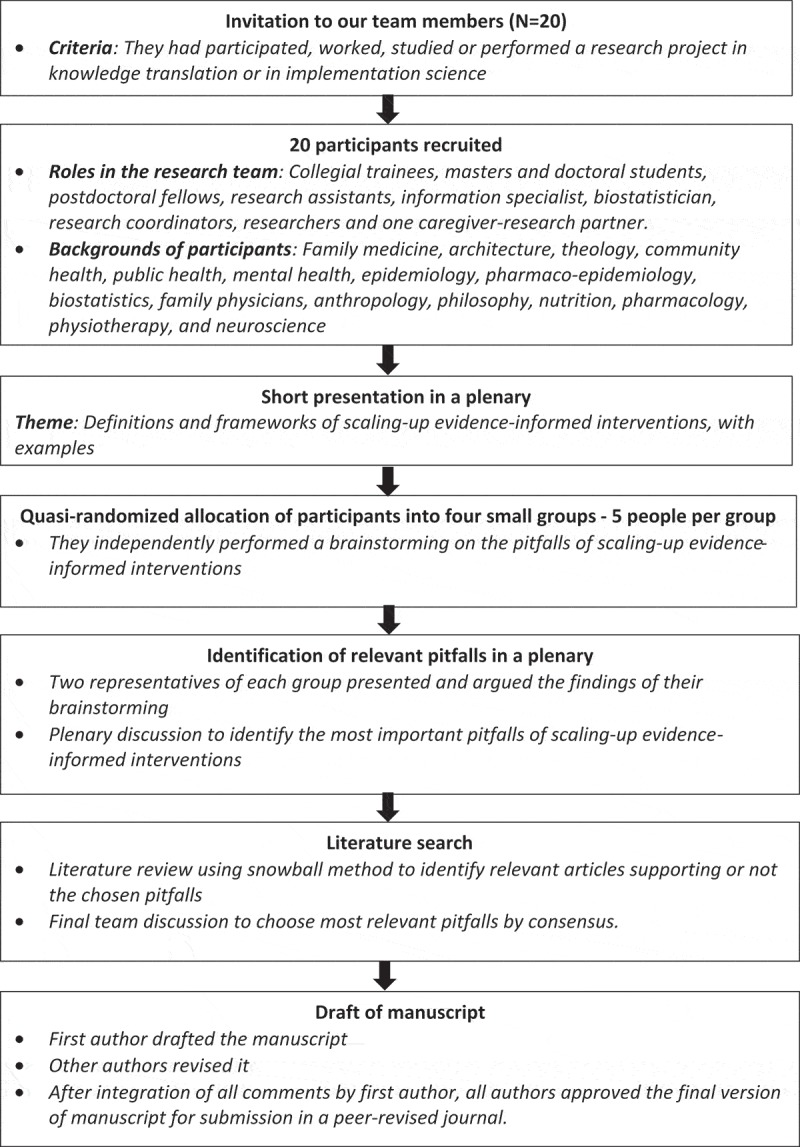
Flowchart of the pitfall identification process.

The members of a multidisciplinary group in implementation science performed a brainstorming on potential pitfalls on the scaling-up of EBIs. This diverse team, including healthcare providers, a scale-up scientist, patient-oriented research specialists, graduate students, postdoctoral fellows, an information specialist, a biostatistician, research assistants, research coordinators, and a caregiver-research partner began to focus on knowledge translation over 15 years ago [21]. They have developed and validated a number of knowledge translation tools (e.g. decision aids, health provider training programs) in the field of shared decision making that they are planning to scale up. To anticipate points of concern, the team was presented with the notion of scaling up and several examples (in low- to high-income countries). They were asked to identify pitfalls they could predict in their own fields (i.e. what elements, in their experiences, readings or observations, could compromise the success of scaling up in those contexts). They debated these and reached consensus on the most important pitfalls. They then performed a literature scan (including low- to high-income countries) by searching PubMed for literature on the identified pitfalls using the snowball approach and a search of the grey literature (Google Scholar and WHO websites). Finally, they met again to discuss the literature and select the most relevant pitfalls (see Figure 1).

This debate paper is meant as a stimulant to further discussion on the topic of scaling up, as well as an advance warning. Scaling up is still a young science (although an old practice) and we focused on the pitfalls so that as the knowledge develops, researchers can be aware of them early on.

**Figure 2.**
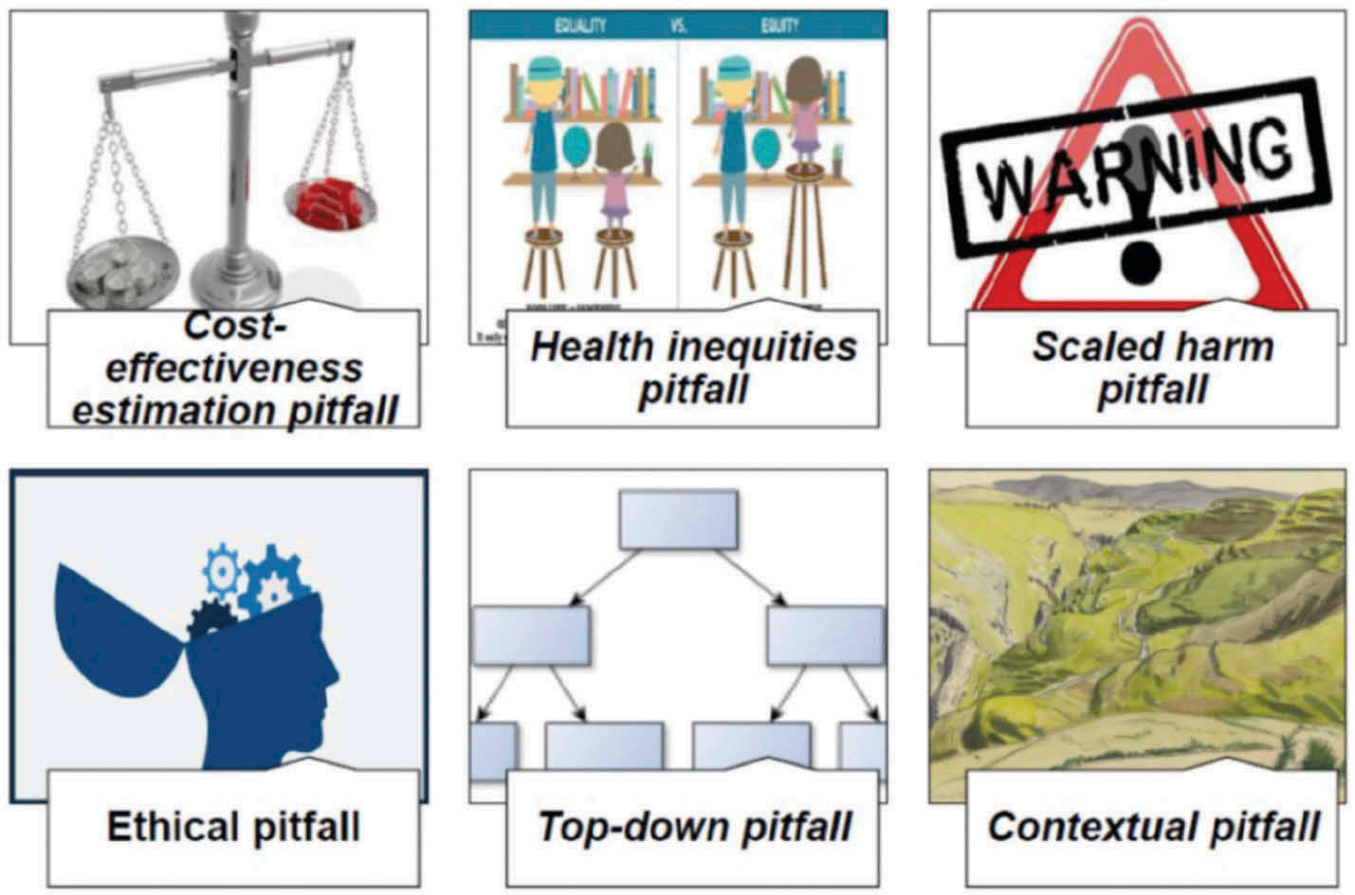
Pitfalls of scaling up evidence-based interventions.

Six potential pitfalls of the scaling-up of EBIs were identified and considered relevant by our multidisciplinary group. They related to cost-effectiveness, equity, harms, ethics, bottom-up/top-down scaling-up, and the context in which the EBI was scaled up (see Figure 2). To discuss them, we identified 45 scaling-up studies that raised these difficulties, of which 13 were on cost-effectiveness estimates or cost-analysis models [22–34], 14 on equity [35–48], four on harms [16,49–52], three on ethics [53–55], six on top-down implementation [42,56–60], and eight on contextual problems [40,43,61–66]. Four out of the 45 studies identified covered more than one pitfall [40,42,43,47].

### The cost-effectiveness estimate pitfall: accurate cost-effectiveness estimates about real-world implementation are almost impossible

One of the incentives for promoting scaling up is the expectation of economies of scale, i.e. a decrease in costs proportionate to increased implementation. For example, the assumption that an increase in patient volume in a hospital service will reduce costs per patient in that service. However, unlike on factory production lines, economies of scale cannot be taken for granted in health services because of their heterogeneity. In the hospital example, increased volume in one service increased the costs per patient of other services in the hospital [67]. Planners may address this heterogeneity by using mathematical models to compare the cost-effectiveness of a variety of EBIs. One study, for example, compared the cost-effectiveness of scaling-up two service models for reducing maternal, fetal, and neonatal deaths in LMICs, and found that scaling-up midwifery services was almost twice as cost-effective as scaling-up obstetrics services [22].

However, cost-effectiveness estimates based on mathematical models are highly complex, contain inherent uncertainties, and depend on numerous assumptions not always based on evidence. A systematic review by Gomez et al. [25] showed that unfounded assumptions about such critical factors as cost, coverage and impact can substantially influence cost-effectiveness estimates. Cost-effectiveness estimates need to consider the size of the targeted population, the incidence or prevalence of the disease or risk factor, the significance of the intervention’s efficacy, and the amount spent or available [29].

Thus while many modelling studies show scaling-up EBIs to be cost-effective [22–24,26,27,30–34], they may not reflect the multi-factorial complexity of the real world. Categorical universal statements about the cost-effectiveness of scaling-up are thus difficult to justify. Further methodological work is needed to better approximate the cost-effectiveness of scaling-up EBIs.

### The health inequities pitfall: some people will necessarily be left out

Many studies highlight equity as a motive for scaling-up effective healthcare interventions: an EBI that is delivered only to a small population constitutes a health inequity, as others are deprived of its proven health benefits [36,38–40,42,43,46–48].

Others point out the importance of ensuring the process of scale-up itself occurs in an equitable way [36,38–40,42,43,46–48], notwithstanding the challenge this represents [37,38,40,45]. A study in Zambia, for example, used equity measures when evaluating a scaling-up model for integrating HIV/AIDS treatment into primary care services [46]. The process not only improved the efficient use of staff time and clinic space but also the equitable delivery of care to HIV/AIDS and non-HIV/AIDS patients alike [46].

However, ensuring equitable access to the EBIs for a target population that includes poor and vulnerable groups, while maintaining its quality within a given budget, can be difficult [35,41], especially because sufficient material, financial and/or human resources are rarely available to these groups [44,45]. For example, during a similar campaign to scale up the distribution of insecticide-treated mosquito nets Africa-wide, equitable access to malaria treatment was reached in only 30% of studied countries, and the urban and richest quintile of households were the beneficiaries in most cases [45]. Considerations of equity must be integrated into scaling-up strategies and health equity metrics need further exploration.

### The scaled-up harm pitfall: harms as well as benefits may be amplified by the scaling-up

Scaling-up EBIs risks amplifying their harms as well as their benefits [49–51]. For example, in their study about scaling-up male circumcision in the context of HIV prevention, Kilima et al. reported severe bleeding, delayed wound healing, and wound sepsis as the most frequent adverse effects [49]. These are the known risks of male circumcision, but they were amplified by the scaling-up. Indeed, the risks were disproportionally increased in the larger targeted population because of insufficient equipment or skills for sterilizing the circumcision tools, inadequate training of the healthcare providers, and lack of resources for monitoring the circumcised persons.

Evidence of the risks, feasibility and acceptability of an intervention in one context may not be sufficient to support its implementation on a wider scale [52]. Health authorities planning to scale up EBIs must explicitly predict harmful effects and risks, closely monitor them at the implementation phase, and take immediate steps to reduce or mitigate them.

### The ethical pitfall: informed consent may be a challenge on a grander scale

Ensuring that all ethical requirements, such as informed consent, are met at the population level can be a challenge in scaling-up EBIs. For example, the United Nations Programme on HIV/AIDS and the WHO recommended in 2004 that for scaling-up ethical HIV/AIDS testing: 1) the results must be confidential; 2) the intervention must be accompanied by an appropriate counseling; and 3) consent to be tested must be given in an informed, specific and voluntary way by the person to be tested [53,55]. However, counselling and informed consent are slow and costly, and in reality may slow down the achievement of universal access to testing and to urgently needed treatment.

Sometimes, indeed, scaling-up itself is the ethical imperative, for example, the scale-up of community mental health services in countries where families and health professionals sometimes resort to deception, coercion and restraints in caring for people with debilitating mental illnesses. Yet scaling-up effective mental health services is difficult in a situation where resources are limited, the human rights of the mentally ill are routinely denied, and the legislative framework is weak [54].

### The top-down pitfall: the needs, preferences and culture of end-users may be forgotten

When scaling-up takes place as a result of a decision from above, population-wide interventions may not reflect the specific needs, preferences, or values of the targeted end-users. Local communities may be disempowered, and the distance (geographical and/or cultural) between policy-makers, health authorities and those implementing the EBI on the ground may result in deformation of the original EBI. The alignment of EBIs with local end-user priorities and contexts, or a bottom-up approach, is essential for the success of scaling-up [47,57,59,60,68]. On the other hand, a top-down approach to scaling-up is likely to have more financial resources at hand and to prioritize interventions that address population-level risk factors and diseases. In addition, political will is a main facilitator of scaling-up EBIs [47,57,59,60,68]. EBIs that are scaled up using a bottom-up approach, which usually involves communities, patients and participatory methodologies, may lose their effectiveness as they demand a certain level of local engagement which is difficult to replicate at scale. However, some eras and political climates are more favorable for this kind of approach, such as the scaling up of the Jyorei community health insurance system in nineteenth-century Japan [42].

Ultimately, both bottom-up and top-down approaches seem to be necessary to optimize the success of scaling-up EBIs [58]. Those responsible for scaling-up EBIs need to collaborate closely with policy-makers and system managers as well as maintain close association with end-users such as clinicians, patients, caregivers and communities.

### The contextual pitfall: inadequate knowledge of the context

The contextual factors that affect scaling-up can be social, physical, regulatory, political or economic [62]. They can be at the micro-, meso- or macro- scale. Lack of knowledge or understanding of these factors can be a major obstacle to effective scaling up [43,61,62,64–66]. At the micro-scale, for example, Chopra et al. assessed the infant feeding components of a scaled-up program to prevent mother-to-child HIV transmission in four African countries [61]. They observed that health workers almost universally believed (mistakenly) that an HIV positive mother who breastfeeds will always infect her child and that breastfeeding avoidance by a mother indicates she is HIV positive [61]. Providing additional financial resources may not be enough to change healthcare providers’ old clinical habits, beliefs, or their personal motivation to adopt new behaviors. The lack of the required number of qualified human resources to conduct the intervention can also limit scaling-up [40]. On the meso-level, scaling-up an EBI successfully also depends on the health system’s capacity to effectively deliver the EBI, including having the appropriate infrastructure, management, and leadership to make it possible [63]. And on the macro-level, success depends on efforts by regulatory, political and economic sectors to ensure the feasibility of the process [66]. Ultimately, it may not be possible to adapt the EBI to every context.

## Conclusion

Scaling-up can be a powerful process for reducing evidence-practice gaps and spreading the benefits of EBIs to those who most need them, but there are significant pitfalls. Although this study did not survey the entire literature on scaling-up, it showed that in designing scale-up studies, investigators need to develop rigorous methods for addressing pitfalls related to cost-effectiveness, equity, ethical standards, amplification of potential harms, top-down implementation, and contextual appropriateness.

## Paper context

This current debate paper is written in a context in which policy-makers worldwide are increasingly interested in scaling up evidence-based interventions (EBIs) to larger populations. Although the scaling-up of EBIs has the great potential to reduce the gap between EBIs and practices, major pitfalls need to be anticipated. This paper discusses these pitfalls to permit to investigators to better plan their scaling-up of EBIs.
